# Catalase Overexpression Drives an Aggressive Phenotype in Glioblastoma

**DOI:** 10.3390/antiox10121988

**Published:** 2021-12-14

**Authors:** Susanne Flor, Claudia R. Oliva, Md Yousuf Ali, Kristen L. Coleman, Jeremy D. Greenlee, Karra A. Jones, Varun Monga, Corinne E. Griguer

**Affiliations:** 1 Free Radical & Radiation Biology Program, Department of Radiation Oncology, University of Iowa, Iowa, IA 52242, USA; susanne-flor@uiowa.edu (S.F.); claudia-oliva@uiowa.edu (C.R.O.); 2 Interdisciplinary Graduate Program in Human Toxicology, Department of Radiation Oncology, University of Iowa, Iowa, IA 52242, USA; ali-mdyousuf@uiowa.edu; 3 Holden Comprehensive Cancer Center, University of Iowa, Iowa, IA 52242, USA; kristen-coleman@uiowa.edu; 4 Department of Neurosurgery, University of Iowa, Iowa, IA 52242, USA; jeremy-greenlee@uiowa.edu; 5 Department of Pathology, University of Iowa, Iowa, IA 52242, USA; karra-jones@uiowa.edu; 6 Department of Internal Medicine, University of Iowa, Iowa, IA 52242, USA; varun-monga@uiowa.edu

**Keywords:** catalase, glioblastoma multiforme, reactive oxygen species (ROS), hydrogen peroxide, radiation, temozolomide

## Abstract

Glioblastoma remains the deadliest form of brain cancer, largely because these tumors become resistant to standard of care treatment with radiation and chemotherapy. Intracellular production of reactive oxygen species (ROS) is necessary for chemo- and radiotherapy-induced cytotoxicity. Here, we assessed whether antioxidant catalase (CAT) affects glioma cell sensitivity to temozolomide and radiation. Using The Cancer Genome Atlas database, we found that CAT mRNA expression is upregulated in glioma tumor tissue compared with non-tumor tissue, and the level of expression negatively correlates with the overall survival of patients with high-grade glioma. In U251 glioma cells, CAT overexpression substantially decreased the basal level of hydrogen peroxide, enhanced anchorage-independent cell growth, and facilitated resistance to the chemotherapeutic drug temozolomide and ionizing radiation. Importantly, pharmacological inhibition of CAT activity reduced the proliferation of glioma cells isolated from patient biopsy samples. Moreover, U251 cells overexpressing CAT formed neurospheres in neurobasal medium, whereas control cells did not, suggesting that the radio- and chemoresistance conferred by CAT may be due in part to the enrichment of glioma stem cell populations. Finally, CAT overexpression significantly decreased survival in an orthotopic mouse model of glioma. These results demonstrate that CAT regulates chemo- and radioresistance in human glioma.

## 1. Introduction

Glioblastoma (GBM) is the most aggressive form of brain cancer, with a mean survival of only 14 months [1], even with the current standard of care treatment, which includes temozolomide (TMZ) combined with radiotherapy [2,3]. Although there is a significant initial benefit, treatment eventually becomes ineffective due to the development of tumor cell resistance. Indeed, resistance to radio-chemotherapy presents the most challenging barrier in the successful treatment of cancer and is one of the main phenomena underlying the failure to achieve a sustainable clinical benefit for patients with GBM [4,5].

Many factors have been associated with GBM resistance to radiation and chemotherapy, including tumor hypoxia, efficient and redundant DNA repair capacities [6], glioma stem cells (GSCs) [7], and increased expression of antioxidant enzymes that reduce the accumulation of reactive oxygen species (ROS) [8,9]. ROS comprises free radicals (superoxide, hydroxyl radical, singlet oxygen) and non-radical species (e.g., hydrogen peroxide [H_2_O_2_]) that activate signaling pathways necessary for cell growth, proliferation, and differentiation [10,11]. However, excess cellular levels of ROS cause damage to important macromolecules, such as DNA, proteins, and lipids [12], which can lead to the activation of cell death signaling pathways [13,14]. Intracellular ROS levels are controlled by a complex network of antioxidant enzymes (e.g., superoxide dismutase and glutathione peroxidase) [15].

CAT is a key enzyme in the metabolism of H_2_O_2_. The molecular mechanisms regulating the expression of catalase (CAT) are not completely understood. Altered expression levels of CAT have been reported in tumor tissues. Specifically, the downregulation of CAT expression has been shown in pancreatic cancer cells and mouse skin tumors [16,17], but upregulation of CAT expression has been reported in melanoma [18], colon cancer [19], gastric adenocarcinoma [20], and GBM [21] cells. Importantly, altered CAT expression has been correlated with resistance to therapy in GBM [22,23], pancreatic cancer [9], and breast cancer cells [24]. In our previous studies, we demonstrated that glioma cell resistance to TMZ-induced oxidative stress is mediated by a reduction in the levels of mitochondrial ROS and enhanced antioxidant production and is associated with increased CAT activity [21,25,26].

Although studies by us and others have indicated an inverse correlation between the level of CAT expression or activity and resistance to therapy in different cancer cells, other studies have shown divergent results. In particular, controversy remains as to whether CAT is up- or downregulated in gliomas [22,25,26,27,28]. Thus, it is important to understand the precise roles of CAT in GBM biology and resistance to therapy. In this study, we investigated the association between CAT expression in GBM tumors and patient survival, as well as the cellular and molecular mechanisms by which CAT influences therapeutic resistance in GBM.

## 2. Materials and Methods

### 2.1. Gene Expression Analyses

For comparisons between high-grade glioma and normal brain samples, CAT read counts of non-tumor (28 samples), mixed glioma (11 samples), oligodendroglioma (67 samples), astrocytoma (147 samples), and GBM (219 samples) were obtained from TCGA. Evaluation of TCGA glioma patient gene expression data and survival analysis were performed with the TCGA Rembrandt dataset. For Kaplan-Meier survival analysis, median gene expression was selected as the cutoff to split high and low expresser populations. The TCGA database can be downloaded from the GlioVis data portal (http://gliovis.bioinfo.cnio.es/, date accessed 8 September 2021).

### 2.2. Cell Culture and Electroporation

Glioma cells were cultured as we previously described [21,29,30]. Cells were electroporated using a Gene Pulser Xcell Electroporation System (BioRad, Hercules, CA, USA) under the following conditions: square wave pulse, 25 msec, and 140V. U251 cells were electroporated with CMV6 plasmids containing Myc-DDK-epitope-tagged human CAT or the pCMV6-Entry mammalian vector with C-terminal Myc-DDK Tag as the control (Catalog # RC210763 and PS100001, respectively; OriGene Technologies, Rockville, MD, USA). To generate cell lines stably overexpressing CAT, cells were selected with G418 (800 µg/mL) for 15 days. The stable lines isolated were characterized for the level of CAT by Western blot analysis and enzymatic activity.

### 2.3. CAT Activity

CAT activity was determined as previously described [28]. Briefly, cells were plated in 100-mm tissue culture plates and grown to 70–75% confluence. Cells were washed twice in phosphate buffered saline (PBS) and then collected and lysed in 50 mM potassium phosphate buffer (PB) pH 7.0 via sonication. For each sample, 100 µg of cell extract was added to 30 mM H_2_O_2_ in 50 mM PB. H_2_O_2_ consumption was measured at 240 nm for 180 s (recorded at 15-s intervals) using a Beckman DU 800 UV spectrophotometer (Beckman Coulter, Brea, CA, USA) at 37 °C. For pharmacological inhibition of CAT, cells were treated with 3-AT or vehicle (DMSO) for 24 h. Cells were then washed in PBS and used for subsequent experiments.

### 2.4. Acquisition of Tissue Specimens and Tumor Dissociation

The protocol for this study was approved by the Institutional Review Board for Human Use at the University of Iowa (IRB 201103721). Patients with high-grade gliomas were identified by the University of Iowa, Tissue Procurement Core for inclusion in the Glioma Library Project. All patients provided written informed consent for the surgical procedures and gave permission for the use of resected tissue specimens, and all samples were de-identified to maintain confidentiality. Tumor tissues were dissociated into single-cell suspensions using a Brain Tumor Dissociation Kit (Miltenyi Biotec Inc., Auburn, CA, USA) according to the manufacturer’s protocol. Single-cell suspensions were cultured in a neurobasal medium as we previously described [21,31]. Cell stocks were stored in liquid nitrogen until use.

### 2.5. Measurement of Intracellular ROS

The generation of H_2_O_2_ was measured using 10 μM Amplex^TM^ Red (ThermoFisher Scientific, Waltham, MA, USA, Cat. # A12222) in the presence of 38 units/mL superoxide dismutase (SOD; Sigma, St. Louis, MO, USA, CAT. # S9697) and 5 units/mL horseradish peroxidase (HRP; Sigma, St. Louis, MO, USA, Cat. # P8375). The fluorescent signal generated by the oxidation of the probe was measured using a FluoroMax-3 spectrofluorometer (λemission = 565 nm, λexcitation = 587 nm, Horiba Jobin Yvon, Edison, NJ, USA). The fluorescence values of each experiment were converted to H_2_O_2_ concentration using a standard curve and expressed as pmoles/min/10^5^ cells.

### 2.6. Cell Proliferation and Anchorage-Independent Clonogenic Assays

For cell proliferation, cells were seeded into 6-well plates (2 × 10^4^ cells/well). Cell number was assayed every 24 h for 5 days using a TC20 automated cell counter (BioRad, Hercules, CA, USA). For crystal violet cell proliferation assays [32], cells were fixed with ice-cold 3.7% paraformaldehyde, washed twice with PBS, and stored at −80 °C overnight. Cells were then stained with 0.05% CV solution and air-dried. The dye was solubilized with 10% acetic acid, and absorbance was measured at 590 nm. Anchorage-independent clonogenic assays were performed as we previously described [21,33].

### 2.7. Xenograft GBM Tumors

All surgical and experimental procedures and animal care practices were performed in compliance with the policies approved by the Institutional Animal Care and Use Committee of the University of Iowa. Establishment of intracranial tumors was performed as we previously described [21,34]. Any animal exhibiting signs of neurological deterioration was killed and the brain removed for examination. Paraffin-embedded tumor tissues were serially sectioned (5 μm), and sections were counterstained with hematoxylin and eosin (H&E, Abcam, Waltham, MA, USA, Cat. # ab245880), as we previously described [21].

### 2.8. Clonogenic Survival Assays

Clonogenic survival assays were performed as previously described [35]. Briefly, cells were plated and allowed to attach for 24 h, then treated with 50–250 µM TMZ or 0.5% DMSO (vehicle control) for 48 h followed by irradiation (2–8 Gy) at a dose rate of 0.65 Gy/min^−1^ using a 6000 Ci^137^Cs source. Cells were trypsinized immediately after treatment, counted, and seeded into 6-well plates at varying densities. The dishes were maintained in an incubator at 37 °C for 10 days to allow colony formation. The colonies were then fixed with 70% ethanol, stained with Coomassie blue, and counted (colonies containing > 50 cells were scored). Plating efficiency was determined by the following formula: (number of colonies formed/number of cells inoculated) × 100.

### 2.9. Cell Cycle Analysis

Cell cycle analysis was performed as we previously described [34]. Briefly, cells were treated with DMSO (control) or 300 μM TMZ for 48 h, then washed with PBS, trypsinized, and resuspended in PBS with 0.1% TritonX-100. PI and ribonuclease A were added at a final concentration of 10 μg/mL. Cells were analyzed by flow cytometry with a BD FACScalibur for DNA content, and the percentage of cells in the G1, S, and G2/M phases was established using ModFit LT (Verity Software House, Topsham, ME, USA).

### 2.10. Determination of Apoptosis

The apoptotic response after treatment with 250 μM TMZ or 10 μM staurosporine (positive control) was measured by flow cytometry as we described previously [29,30]. Treated and untreated cells were harvested, washed once with PBS, and stained with an Annexin V-PE Apoptosis Detection Kit (BD Pharmigen, San Diego, CA, USA) according to the manufacturer’s instructions. Samples were analyzed by flow cytometry using a BD LSR II flow cytometer using λ Ex. 492–495 nm, λ Em. 517–527 nm. For each sample, 10,000 cells were analyzed, and the number of apoptotic cells was calculated with FlowJo Software Version 10 (FlowJo, LLC, Ashland, OR, USA).

### 2.11. In Vitro Limiting Dilution Assay

In vitro dilution assays were performed as we previously described [21,34,36]. Briefly, cells were plated at 1, 2, 5, 10, 25, 50, 100, and 200 cells per well in 96-well plates in the presence or absence of 3-AT (25 mM). Ten days after plating, the number of neurospheres in each well and the percentage of positive wells were quantified by manual counting. Extreme limiting dilution assay analyses were performed on the data as we previously described [21].

### 2.12. Statistics

Data were evaluated using GraphPad. Differences among the groups were tested using either unpaired two-tailed *t*-test or one- or two-way analysis of variance (ANOVA) followed by Tukey’s multiple comparison test, and probability (*p*) values were reported, with *p* < 0.05 indicating statistical significance. Experiments were performed in duplicate or triplicate and repeated twice or more to verify results. Data are expressed as the mean ± standard deviation (SD) and significance indicated as follows: *p* < 0.05 (*), *p* < 0.01 (**), *p* < 0.001 (***), and *p* < 0.0001 (****), respectively.

## 3. Results

### 3.1. Overexpression of CAT in Malignant Brain Tumors

**Figure 1:**
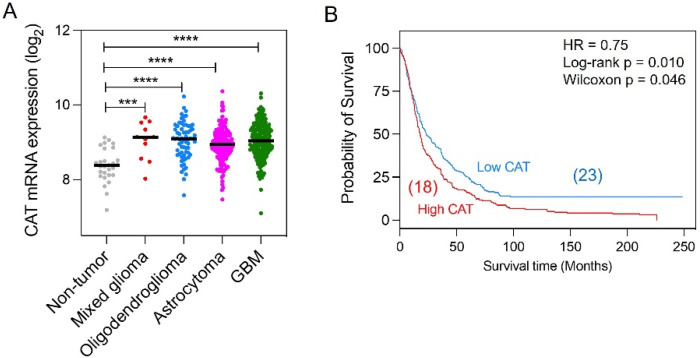
Expression of CAT mRNA in human glioma. (**A**) Expression of CAT mRNA in glioma tissue and non-tumor brain tissue samples from the Rembrandt dataset. Median values are represented as lines in the scatter plot. *** *p* < 0.001, **** *p* < 0.0001 by ANOVA. (**B**) Kaplan-Meier survival curves of overall survival in patients with glioma stratified by CAT mRNA expression levels in tumors (low CAT, *n* = 208; high CAT, *n* = 189). Numbers between brackets indicate median survival time. CAT, catalase.

We previously reported that CAT activity is elevated in TMZ-resistant glioma cells [21]. To investigate if CAT expression is clinically relevant in gliomas, we interrogated The Cancer Genome Atlas (TCGA) data accessed via GlioVis [37]. We compared CAT mRNA levels in glioma tumor tissue to levels in control brain tissues in datasets from Rembrandt cohorts comprising results from 537 samples. Compared with the expression in non-tumor tissue, CAT mRNA expression was significantly upregulated in astrocytoma, oligodendroglioma, mixed glioma, and GBM samples (Figure 1A, Supplementary Table S1). Analysis of CAT mRNA in glioma samples of different grades showed that the level of CAT mRNA expression in the tumor correlated inversely with overall survival (OS) in patients (high median CAT expression: OS = 18.0 months; low median CAT expression: OS = 23.4 months; *p* = 0.0107 by log-rank test) (Figure 1B). Together, these results confirm that human glioma tumors express high levels of CAT, and higher tumor expression of CAT is associated with poor prognosis.

### 3.2. Stable Overexpression of CAT Results in Decreased Levels of Intracellular H_2_O_2_

**Figure 2:**
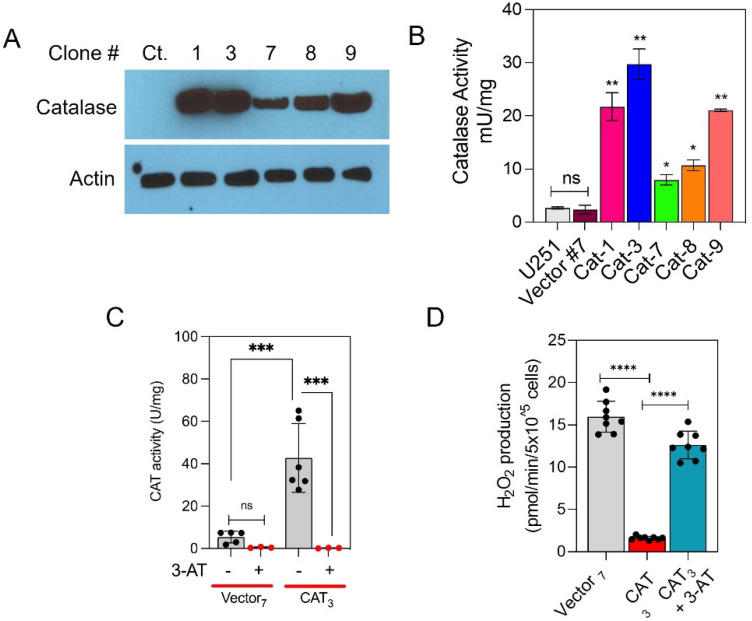
CAT expression and activity in stably transfected glioma cells. U251 cells were transfected with a CATexpressing (pCMV6−CAT−-Myc−DDK) or control vector (pCMV6−Myc−DDK) construct and treated with G418 to produce stably transfected clones. (**A**) Representative Western blots depicting CAT protein expression in lysates of select clones. Actin was used as a loading control. (**B**) Quantitative analysis of mean CAT activity in the same clones. (**C**) Quantitative analysis of mean CAT activity in control or CAT-overexpressing U251 cells treated without or with the CAT inhibitor 3−amino−1,2,4−triazole (3−AT; 25 mM for 24 h) in select clones. (**D**) Bar graph showing the rate of H_2_O_2_ production as pmoles/min/10^5^ cells determined using the fluorescent probe AmplexRed. Graphs represent mean ± SEM from duplicate determinations from three independent experiments. * *p <* 0.05, ** *p* < 0.01, *** *p* < 0.001, **** *p <* 0.0001 calculated by Student’s *t*-test.

To evaluate the effect of CAT overexpression on glioma phenotype, we stably transfected U251 glioma cells with a vector encoding CAT cDNA. U251 cells stably transfected with an empty vector were used as the control. CAT protein expression was verified by Western blot analyses (Figure 2A) and assays for enzymatic activity (H_2_O_2_ consumption) (Figure 2B). CAT expression in parental and vector-transfected cells is often weak or undetectable by western blot under the tested conditions, confirming the low abundance of CAT in U251 cells. We selected U251 clone 3, which exhibited CAT activity 15 times higher than that in control cells, for further analysis. Treatment with an irreversible inhibitor of CAT [38], 3-amino-1,2,4-triazole (3-AT; 25 mM for 24 h), completely abrogated CAT activity in the CAT-overexpressing cells, demonstrating that the difference in CAT activity between the two cell types is due to CAT overexpression (Figure 2C). To assess whether CAT overexpression contributes to ROS scavenging, cellular levels of H_2_O_2_ were measured by monitoring the oxidation of Amplex Red using a spectrofluorometer (FluoroMax-3; Horiba Jobin Yvon, Edison, NJ, USA). As shown in Figure 2B, overexpression of CAT induces a 10-fold decrease in H_2_O_2_ production compared with H_2_O_2_ formation by vector-transfected cells (1.61 ± 0.07 and 15.97 ± 0.65 pmoles/min/10^5^ cells, respectively). Treatment with 3-AT (25 mM, 24 h) increases H_2_O_2_ production in CAT cells (12.63 ± 0.58 pmoles/min/10^5^ cells), indicating that H_2_O_2_ depletion is due to CAT overexpression (Figure 2D).

### 3.3. CAT Overexpression Promotes Cell Proliferation

**Figure 3:**
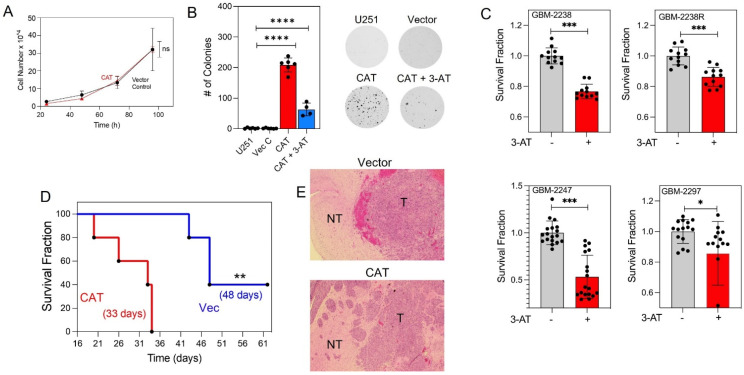
(**A**) Quantitative analysis of cell proliferation over time in adherent cultures of control and CAT-overexpressing U251 cells. (**B**) Quantitative analysis and representative images of anchorage-independent colony formation assays in control and CAT-overexpressing U251 cells cultured in the absence or presence of 3-AT (25 mM). (**C**) Quantitative analysis of proliferation in GSCs isolated from GBM patient biopsy samples and cultured in the presence or absence of 3-AT (25 nM). Data were pooled from three independent experiments. * *p <* 0.05, ** *p* < 0.01, *** *p* < 0.001, **** *p <* 0.0001, ANOVA. (**D**) Kaplan-Meier survival curves of overall survival in nude mice harboring orthotopic brain tumors generated by inoculation with control (*n* = 5) or CAT-overexpressing (*n* = 5) U251 cells (Log-rank test *p* = 0.0027; Wilcoxon test *p* = 0.0043). (**E**) Representative images of tumors resulting from intracranial implantation of vector-control and CAT-overexpressing cells and stained for Hematoxylin and Eosin. Vec, vector; T, tumor; NT, normal tissue.

Since ROS activates signaling pathways that contribute to the regulation of cell proliferation [25], we investigated whether CAT overexpression in glioma cells is associated with a change in cell proliferation. When adherent cell growth was assessed, the doubling time did not differ between CAT overexpressing cells and control U251 cells (18.0 ± 5.7 h and 21.5 ± 4.1 h, respectively; *p* = 0.68) (Figure 3A). However, CAT overexpression markedly promoted colony formation in the anchorage-independent soft agar condition. In fact, colonies were present only in CAT-overexpressing cell cultures (209 ± 23) (Figure 3B), supporting the notion that CAT can facilitate anchorage-independent growth. Treatment with 3-AT caused a 70% reduction in the number of colonies (63 ± 21) (Figure 3B), suggesting that the decrease in intracellular ROS levels caused by CAT overexpression is tightly associated with the increase in soft agar colonies. To assess the clinical relevance of this finding, we investigated the effect of CAT inhibition on the proliferation of GSCs freshly isolated from human GBM samples. As shown in Figure 3C, treatment with 3-AT significantly inhibited cell proliferation in all patient-derived GSC lines tested. To determine if CAT expression influences host survival, CAT-overexpressing or control U251 cells were implanted orthotopically into the brains of nude mice. Median survival was significantly shorter in mice bearing CAT-overexpressing tumor cells (33 days versus 48 days in mice bearing control tumor cells [*p* = 0.0027]) (Figure 3D). Notably, mice bearing CAT cells developed invasive tumors characterized by multifocal lesions throughout the brain parenchyma. In comparison, brains with vector-U251 tumors displayed only single lesions (Figure 3E). Overall, these results suggest the pivotal role of CAT in promoting glioma tumorigenicity and anchorage-independent growth, a hallmark of the aggressive GBM phenotype.

### 3.4. CAT Promotes Resistance to TMZ and Radiation

**Figure 4:**
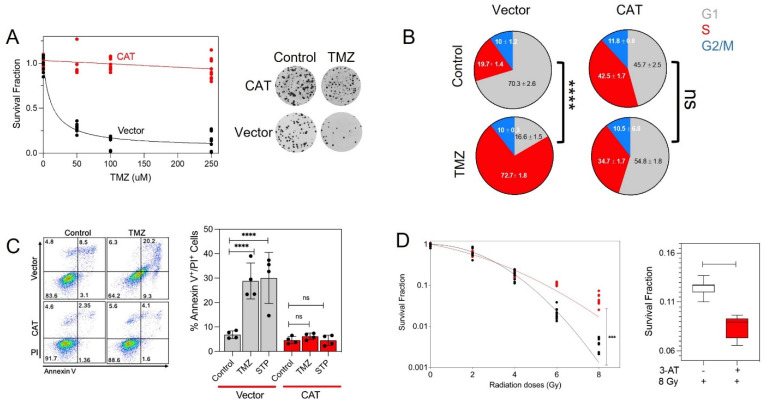
Effect of CAT overexpression on TMZ and radiation resistance in glioma cells. (**A**) Quantitative analysis and representative images of clonogenic survival assays with control and CAT−overexpressing U251 cells treated with various concentrations of TMZ. (**B**) Cell cycle distribution of cells treated with 300 μM TMZ. Numbers indicate the percentage of cells in each phase of the cell cycle, as indicated. Graphs represent mean ± SEM from duplicate determinations from three independent experiments. *** *p* < 0.001, **** *p <* 0.0001 calculated by Student’s *t*-test. (**C**) Determination of apoptotic cells in control and CAT−overexpressing U251 cells after treatment with 250 μM TMZ or 10 μM staurosporine (STP), a positive control for apoptosis. Representative flow cytometry plots obtained in cells treated with DMSO (control) or TMZ and stained for propidium iodide (PI) and annexin V (left); quantitative analysis of flow cytometry results (right). Columns represent the average from triplicate determinations of the percentage of apoptotic cells. (**D**) Quantitative analysis of clonogenic survival assays with control and CAT−overexpressing cells exposed to different radiation doses (**left**); survival fraction of CAT−overexpressing cells exposed to 8 Gy after pretreatment with 3−AT (25 mM) (**right**).

Because the standard of care treatment for GBM involves TMZ as a chemotherapy agent, we used the clonogenic assay to investigate the in vitro long-term survival of glioma cells after TMZ treatment. TMZ had a clear dose-dependent cytotoxic effect on control U251 cells (Figure 4A); these doses were comparable in efficacy to TMZ’s known cytotoxicity [29]. However, the cytotoxic effect of TMZ at any dose was mostly abolished in CAT-overexpressing U251 cells (cell survival rate with 250 µM TMZ, 93% and 10% for CAT-overexpressing and control cells, respectively) (Figure 4A). To evaluate the effect of TMZ on cell proliferation, we used flow cytometry to measure cell cycle progression in CAT-overexpressing and control cells treated with 300 μM TMZ for 48 h. TMZ treatment led to S phase accumulation that reached 72.7% (*p* < 0.0001) at the expense of G_1_ accumulation (16.6%) in control cells but did not affect cell cycle distribution in CAT-overexpressing cells (Figure 4B). We also examined the induction of apoptosis using annexin V/propidium iodide (PI) staining and flow cytometry. Treatment with 300 μM TMZ for 48 h caused a 30% increase in the number of annexin V/PI-positive control cells but did not affect the number of annexin V/PI-positive CAT-overexpressing cells (Figure 4C). Interestingly, CAT-overexpressing cells were also resistant to treatment with staurosporine (10 µM) (Figure 4C), a well-known inducer of apoptosis [39], suggesting that CAT-overexpressing glioma cells are resistant not only to TMZ but to apoptotic stimuli in general.

There is unequivocal pre-clinical and clinical evidence that ROS influence the genotoxic stress caused by ionizing radiation [40]. Given the important role of ROS production in mediating glioma cell sensitivity to radiation, we next examined the effect of radiation on clonogenic survival in the context of CAT overexpression. Treatment with radiation (2, 4, 6, or 8 Gy) dose-dependently decreased the survival fraction in control cells, but the effect at 4, 6, and 8 Gy was markedly diminished in CAT-overexpressing cells (*p* < 0.0001) (Figure 4D). Pretreatment of CAT-overexpressing cells with 3-AT reduced the clonogenic survival after treatment with radiation (*p* < 0.0001) (Figure 4D), further suggesting that CAT may protect glioma cells against radiation-induced toxicity.

### 3.5. CAT Promotes Neurosphere Formation

**Figure 5:**
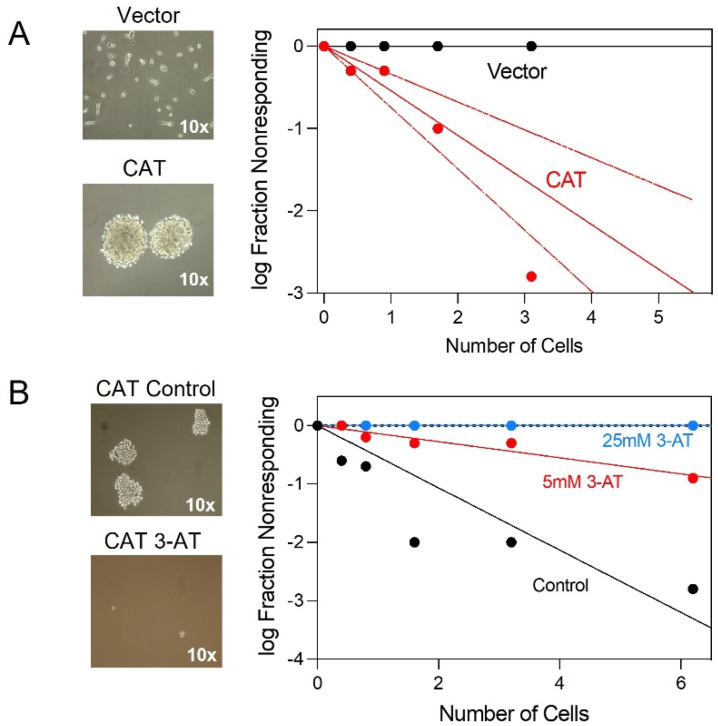
Effect of CAT overexpression on neurosphere formation. In vitro limiting dilution assays of (**A**) vector and CAT expressing cells; (**B**) CAT expressing cells in the presence or absence of 3−AT. Results represent the average from two independent experiments.

Because we previously demonstrated that GSCs display higher CAT expression than non-tumor brain cells [21], we investigated whether CAT-overexpressing U251 cell cultures are enriched in GSCs when cultured in a neurobasal medium supplemented with epidermal growth factor (EGF) and basic fibroblast growth factor (bFGF). CAT-overexpressing cells formed neurospheres ranging from 0.1 to 1.0 mm in diameter over the course of 15 days. In contrast, control cells failed to form neurospheres and attached to the culture dish (Figure 5A). Furthermore, when plated in an in vitro limiting dilution assay, CAT-overexpressing cells formed neurospheres, but control cells did not (Figure 5B). Treatment with 3-AT significantly reduced the frequency of GSCs in CAT-overexpressing cells (1 in 1.3 cells under control conditions versus 1 in 4.6 cells and 1 in 33.5 in cells treated with 5 mM and 25 mM 3-AT, respectively) (Figure 5B). These results suggest a critical role for CAT in mediating glioma neurosphere formation.

## 4. Discussion

Multiple mechanisms have been described to explain therapy resistance in gliomas, including drug inactivation, drug efflux, DNA damage repair, cell death inhibition, tumor hypoxia, and increased expression of antioxidant enzymes [41,42]. We previously demonstrated that TMZ-resistance in glioma cells is due to more efficient mitochondrial coupling and reduced ROS production. Specifically, under conditions of oxidative stress, TMZ-resistant glioma cells generate substantially less ROS and more antioxidant enzymes than TMZ-sensitive glioma cells [30]. Our previous studies also revealed increased expression of CAT, superoxide dismutase 2 (SOD_2_), and BMI1, a protein related to stemness and therapy resistance, in TMZ-resistant glioma cells [21]. In this study, we further assessed the role of CAT upregulation in glioma cell resistance to therapy and demonstrated that chemo- and radioresistance relies upon the regulation of hydrogen peroxide due, at least in part, to altered expression of CAT.

We found that human glioma cells stably overexpressing CAT express lower levels of intracellular H_2_O_2_ than control cells and are resistant to TMZ and radiation. Interestingly, we did not observe differences in the rate of proliferation of CAT and control cells in adherent culture conditions. However, CAT overexpression is associated with a significant increase in anchorage-independent proliferation, a hallmark of the aggressive cancer phenotype, and enhanced capacity for self-renewal. These effects were abrogated by treatment with 3-AT. Similar differences between adherent cell growth and anchorage-independent growth were previously observed in T98G [43], U87 and LN229 [44], and A172 [45] glioblastoma cells.

In agreement with our results, Smith et al. demonstrated that CAT activity is constitutively elevated in 36B10 rat glioma cells compared with normal astrocytes and has an important role in the resistance to oxidative stress and ionizing radiation [26]. Nanjaiah et al. also reported that activation of N-methyl-D-aspartate receptor (NMDAR) attenuated the intracellular ROS production and significantly enhanced the cell viability in LN18 and U251MG glioblastoma cells by increasing CAT activity. These results suggest that enhanced CAT activity contributes to glioma cell survival under exogenous oxidative stress [46].

Significant overexpression of CAT has been observed in a radioresistant variant clone (RRC) of U251 glioma cells. RRC showed an activation of up to 5-fold of antioxidant enzymes, such as SOD, CAT, glutathione peroxidase (GPX), and glutathione reductase (GR). Compared with parental U251 cells, RRC cells increase the activity of major antioxidant enzymes at times soon after radiation, suggesting a rapid scavenge of ROS to minimize the deleterious effects of radiation [23]. As Lee et al. demonstrated, exposure to radiation upregulated CAT and other oxidant enzymes and increased radio and chemoresistance. Our study demonstrated that overexpression of CAT is, at least in part, responsible for the resistant phenotype.

Significant overexpression of CAT has also been observed in human melanoma biopsy samples [18], colon cancer tissue [19], and gastric carcinoma cells [20]. In contrast, it was reported that the expression of antioxidant enzymes decreases during the progression from the normal pancreas to chronic pancreatitis to pancreatic cancer [16]. Similarly, CAT downregulation has been reported during skin cancer progression [17]. Interestingly, CAT overexpression in MCF-7 human breast cancer cells impaired cell proliferation and sensitized the cells to paclitaxel, etoposide, and arsenic trioxide [47]. The discrepancy between results may be due to cell type-specific responses or variations in experimental conditions. Even though our results clearly support a role for CAT in the mechanism of chemo- and radioresistance, we cannot exclude the possibility that the switch to a therapy-resistant phenotype could be the result of a coordinate effect of multiple antioxidant enzymes. For example, it was recently reported that SOD_2_ expression critically regulates tumorigenesis and the acquisition of TMZ resistance in GBM [48]. Additionally, it has been recently suggested that ROS regulates nuclear epigenetic modifications to promote cell survival and stemness [49,50,51,52]. Even though our results clearly support a role for CAT in chemo- and radioresistance, additional research is required to fully understand the underlying mechanism.

Here we also found that a shorter survival time was associated with orthotopic xenograft mice bearing glioma cell overexpression of CAT. Notably, CAT tumors are invasive and multifocal, while vector cells develop single lesions. Multifocal GBMs are rare and usually associated with worse outcomes compared with single lesion GBMs [53,54], supporting an important role of CAT in the development of more aggressive tumors.

## 5. Conclusions

Overall, this study provides strong evidence suggesting that overexpression of CAT in glioma cells leads to increased resistance to TMZ and radiation, suggesting an essential role of H_2_O_2_ in the molecular mechanism by which these treatments lead to tumor cytotoxicity. As a crucial factor in the acquisition of TMZ and radiation resistance, pharmacological inhibition of CAT activity is a promising strategy for the treatment of malignant gliomas, including highly aggressive GBM tumors.

## Data Availability

The data is contained within the article or Supplementary Materials.

## Supplementary Materials

Gene expression data is available online at http://gliovis.bioinfo.cnio.es/ (accessed on 8 September 2021) and https://www.mdpi.com/article/10.3390/antiox10121988/s1, Table S1, Catalase mRNA expression.
